# Nutritional Health Education in Pregnant Women in a Rural Health Centre: Results in Spanish and Foreign Women

**DOI:** 10.3390/healthcare9101293

**Published:** 2021-09-29

**Authors:** Mercedes de Dios-Aguado, María Teresa Agulló-Ortuño, María Idoia Ugarte-Gurrutxaga, Benito Yañez-Araque, Brígida Molina-Gallego, Sagrario Gómez-Cantarino

**Affiliations:** 1 Primary Health Care No.1. Health Centre, Yepes, Castilla-La Mancha Public Health Service (SESCAM), 45005 Toledo, Spain; mded@sescam.jccm.es; 2 Research Group Nursing, Pain and Care (ENDOCU), UCLM, Av. Carlos III s/n, 45071 Toledo, Spain; Maria.Ugarte@uclm.es (M.I.U.-G.); brigida.molina@uclm.es (B.M.-G.); 3 Faculty of Physiotherapy and Nursing of the Toledo Campus, University of Castilla-La Mancha (UCLM), 45071 Toledo, Spain; mariateresa.agullo@uclm.es; 4 Department of Physical Activity and Sports Sciences, Campus Toledo, University of Castilla-La Mancha, 45071 Toledo, Spain; benito.yanez@uclm.es; 5 Health Sciences Research Unit: Nursing (UICISA: E), Coimbra School of Nursing (ESEnfC), 3004-011 Coimbra, Portugal

**Keywords:** health, education, lifestyle, beliefs, risk factors, pregnancy outcome, foreign and Spanish pregnant women

## Abstract

The dietary behaviour of pregnant women, as well as the socio-cultural conditions in which pregnancy takes place, influence obstetric outcomes. To analyse the influence of socioeconomic factors and dietary habits on obstetric outcomes in Spanish and foreign pregnant women living in a rural environment, a population-based, prospective-observational study in a cohort of Spanish and foreign pregnant women in the town of Yepes, in the province of Toledo, Spain was conducted. Foreign pregnant women are ecodependent on their partners, have secondary education and low socioeconomic level. Spanish pregnant women have secondary education, a medium socio-economic level, live with their partners and are economically independent. Moreover, 85% of Spanish pregnant women gave birth at term and reached a gestational age of 40 ± 1.83 weeks. However, only 55% of foreign pregnant women reached a gestational age of 39.72 ± 2.28 weeks. Through health education, pregnant women in this geographical area of Castilla la Mancha, Spain, adopted bicultural dietary patterns, thus reaching the prescribed diet of 2000 Kcal. Through this diet, both Spanish and foreign pregnant women maintained albumin and plasma protein levels within the established range, with no significant differences in obstetric outcomes among pregnant women in the study.

## 1. Introduction

The development of a new life causes changes in the mother’s physiology and psyche throughout pregnancy, resulting in an increased demand for nutrients [1]. Pregnant women can physiologically increase their blood volume by up to 40–45%, resulting in a 25% decrease in serum protein and albumin concentrations [2]. This fact, together with the increase in protein requirements from the second month of gestation due to the growth of maternal and foetal tissues, obliges women to eat a balanced diet that favours placental quality and achieves optimal intrauterine foetal development. Protein accounts for 15–25% of the total caloric intake of a pregnant woman’s diet [3]. To maintain an optimal protein range, pregnant women should receive health education on the consumption of protein-rich foods, such as eggs, milk and dairy products, meat and legumes [4,5].

Applying current protocols and carrying out adequate nutritional education for optimal maternal and foetal development during pregnancy contributes to the achievement of a healthy eating culture in pregnant women. In this way, health professionals who care for women during pregnancy should promote HE from the first visit, focusing on healthy eating habits and lifestyles, mainly those that are unknown and/or harmful to the pregnant woman. Thus, in each trimester, the pregnant woman undergoes a check-up in which her anthropometric measurements are controlled and a blood test is performed to check the serological values of glucose, total protein and albumin, the latter indicators of nutritional quality [6,7,8,9].

In this regard, the International Federation of Gynaecology and Obstetrics, as well as the National Health Systems of several countries [7,8,9], have developed manuals that clearly explain the recommendations on the quantity and quality of food that women should eat during pregnancy, with the intention of maintaining adequate weight gain at this stage of their lives [6,7,8,9,10].

Nowadays, the lifestyle of the population is influenced by the media, cultural and socioeconomic factors. Therefore, the lifestyle of the pregnant woman is influenced by these circumstances as well as her family environment [11]. In this sense, following the conceptual framework of socioeconomic determinants of social inequalities in health, which specifically for foreign women are gender, ethnicity and territory, which together result in language barriers, less economic independence and job insecurity [12]. Thus, foreign pregnant women are more likely to have an inadequate diet. At the same time, Spanish pregnant women, despite being aware of the benefits of a Mediterranean diet, do not always follow it. This situation should be considered by the health professionals involved in HE from the beginning of the gestational period and continuing throughout the three trimesters of pregnancy. This situation will allow the pregnant woman to acquire adequate nutritional habits during her pregnancy, which will benefit the different members of the family unit. This will facilitate a diet in accordance with nutritional needs during the different stages of life [1,2,3]. HE should be carried out with the aim of reinforcing an adequate diet during pregnancy and achieving optimal obstetric outcomes [3,11,13,14,15,16,17].

The aim of this research is to describe the influence of socioeconomic factors and dietary behaviours on the obstetric outcomes of Spanish and foreign pregnant women living in a rural environment, specifically in the town of Yepes, in the province of Toledo, Spain.

## 2. Materials and Methods

### 2.1. Study Design and Participants

This is prospective observational population-based research conducted in the period from January 2019 to March 2020. The selected sample is pregnant women residing in a rural geographical area of the Community of Castilla La Mancha (Yepes, Toledo, Spain). In this rural area in 2020, according to the municipal census, there were 2570 women, of whom 1040 were of childbearing age (15–45 years), of whom 843 were Spanish and 197 foreign.

The overall research sample is composed of a total of 194 women who were attended throughout their pregnancy in the rural Public Health Centre of Yepes (Toledo). The sample is divided into two groups: (1) pregnant women of Spanish nationality comprising 121 women; (2) pregnant women of foreign nationality comprising 73 women. Inclusion criteria were women over 18 years of age, with a singleton pregnancy, with knowledge of Spanish, English or French and who voluntarily agreed to participate in the study. Exclusion criteria were women with pregestational pathologies, such as hypertension, preeclampsia in previous pregnancies or gestational diabetes.

During the scheduled visits to the Health Centre, all women answered questions (anamnesis) asked by the professionals about their obstetric history, socioeconomic status and dietary habits. During the three trimesters of pregnancy, women had their anthropometric measurements taken on the same scales and scalimeter, and analytical tests were also carried out in the same laboratory. All pregnant women were provided with the 2000 Kcalorie diet implemented by the Obstetrics Service of the Complejo Hospitalario Universitario de Toledo (CHUT).

Information on pregnancy outcomes (type of delivery, hours of duration, birth weight of the newborn) was obtained from the medical records of each patient.

### 2.2. Evaluation of Biochemical Parameters in Blood

Blood samples were taken at each of the scheduled visits for the pregnant women so that determinations were made in the first trimester (T1; between 6 and 9 weeks of gestation), second trimester (T2; 26 weeks of gestation), and third trimester (T3; 35 weeks of gestation).

The blood samples were obtained by venous puncture of the forearm with EDTA collection tubes, immediately centrifuged and transported to the central laboratory for analysis. Metabolites were quantified by commercial enzymatic and standard chemistry methods on an ADVIA^®^2400 Automated Clinical Chemistry System (Bayer Healthcare LL; Whippany, NJ, USA). The reference ranges established in the laboratory are 6.4–8.3 g/dL for total plasma protein, 3.4–4.8 g/dL for plasma albumin, and 70–100 mg/dL for plasma glucose.

The serum parameters of total protein and albumin were chosen for analysis in this study because they are indicators of the nutritional quality of the woman’s diet. In addition, glucose was chosen because it is a recommended indicator in the CHUT Obstetrics Service protocol.

### 2.3. Statistical Analysis

The collected data were all quantitative and the IBM SPSS Statistics 25 software (IBM, Armonk, NY, USA) was used for data analysis. After data collection, the data were transferred to SPSS to create the database needed for the analyses. Once the database was created, those cases that did not meet the previously defined requirements to belong to each population sample were eliminated.

Subsequently, the scores of each subject in the two groups were calculated. Statistics of median, mean, standard deviation (SD) or interquartile range (IR) were used to describe the parameters used. Differences in parameter distributions between the two groups of patients studied were analyzed using Student’s *t*-tests, ANOVA, or Mann–Whitney U test if the distribution deviated from normality. For categorical data, the chi-square test was used and relationships between variables were established with Pearson or Spearman correlation coefficients. All tests were two-tailed and a *p* < 0.05 was considered significant.

### 2.4. Ethical Consideration

This study has been conducted to the highest ethical standards, in accordance with the Declaration of Helsinki, International Conference on Harmonization Guidelines for Good Clinical Practice, and national ethical and legal requirements. All women included in this study signed an informed consent form prior to their inclusion in the study. The Clinical and Research Ethics Committee of the Toledo Hospital Complex approved the objectives and procedures of this study.

## 3. Results

This section may be divided into subheadings. It should provide a concise and precise description of the experimental results, their interpretation, as well as the experimental conclusions that can be drawn.

### 3.1. Socioeconomic Characteristics of the Women Included in the Study

The sample of 194 pregnant women was divided into two groups, with 121 women belonging to the group of Spanish pregnant women and 73 women belonging to the group of foreign pregnant women.

The socio-economic status variable, which combines income, education and employment, can be correlated with the socio-economic status given by the National Statistics Institute (INE), which relates annual household income and average income per consumption unit, which in the case of the municipality of Yepes, was EUR 16,908 per person [18]. Thus, the women in the research can be classified into four categories of socio-economic status: Low, Medium, Medium-high and High. Table 1 shows that 47.9% of Spanish women are in the medium socio-economic level, while 56.2% of foreign women are in the low socio-economic level.

Therefore, the study variables socio-economic level and economic dependence between Spanish and foreign women show a significant statistical difference as *p* =< 0.001.

In this sense, it is shown that 24.7% of foreign pregnant women are economically independent, compared to 55.5% of Spanish pregnant women. Therefore, this variable shows a significant difference between both groups of pregnant women, since *p* =<0.001.

Likewise, there are no significant differences related to their level of education, since 54.5% of Spanish women have secondary education compared to 56.2% of foreign women and 11.0% of foreign pregnant women have university studies compared to 19% of Spanish women.

The average age of Spanish pregnant women is 30.88 years, while in the group of foreign pregnant women it is 30.47 years, so this variable does not show significant differences.

### 3.2. Pregnant Women’s Dietary Behaviour

To address this point, it is necessary to consider that the 73 foreign pregnant women included 7 Romanians, 3 Bulgarians, 25 Moroccans, 14 Algerians, 4 Tunisians, 2 Chinese, 2 Syrians, 6 Bolivians, 6 Peruvians, 2 Argentinians, 1 Colombian and 1 Mexican.

During the first visit with the primary care nurse, the nutrition of the pregnant women in both groups was verified.

Subsequently, the 2000 Kcalorie diet implemented by the CHT Obstetrics Service and the minimum portions to be consumed from each of the food groups were given and explained, adapting them to their beliefs, culinary habits, religion and culture (Table 2).

Adherence to the diet was checked during subsequent visits scheduled for the second and third trimesters.

It is observed that in both groups of pregnant women, between the second and third trimester of gestation, there are positive changes in diet, with an increase in the consumption of dairy products, cereals, pulses, vegetables and fruit. At the same time, there was an increase in the consumption of eggs in both groups.

However, the consumption of meat and fish increased in the group of Spanish pregnant women and no significant changes were found in the group of foreign pregnant women. It should be noted that in both groups there was a decrease in the consumption of precooked food and pastries. This is related to receiving health education and high motivation for change on the part of the pregnant women.

### 3.3. Biochemical Parameters of the Women Included in the Study

Total plasma protein concentration decreased progressively throughout the three trimesters of pregnancy in all women in our cohort. However, we observed statistically significant differences in these levels throughout pregnancy between Spanish and immigrant women (Table 3), with immigrant women maintaining higher protein levels. This is similar with respect to plasma albumin levels, although there are no statistically significant differences between the two population groups.

With respect to plasma glucose measurements, none of the women included in this study had values indicative of gestational diabetes. However, the glucose levels of the immigrant women were slightly higher than those of the Spanish women. This was statistically significant in the third trimester (*p* = 0.042, Table 3).

### 3.4. Anthropometric Characteristics and Obstetric Data of the Women Included in the Study

In the study, at the beginning of pregnancy, women in the foreign group had a higher pregestational weight than women in the Spanish group. However, this difference was not found at the end of pregnancy, with a similar BMI between both population groups. Only 4.12% of pregnant women were underweight at the beginning of pregnancy (BMI < 18.5 kg/m^2^), and all of them were of Spanish nationality. In contrast, 41.24% of the women started their pregnancy overweight (BMI > 25 kg/m^2^), of whom 44 were Spanish and 36 were of foreign nationality (2 Romanian, 12 Moroccan, 6 Bolivian, 4 Peruvian, 6 Algerian, 2 Tunisian, 2 Argentinean, 1 Bulgarian and 1 Mexican) (Table 4).

The mean height of the Spanish pregnant women was 1.62 m and 1.63 m for the foreign pregnant women. Relating the type of delivery to this variable, it was observed that 19.2% of Spanish pregnant women had a caesarean delivery, mainly associated with women with a height of 1.58 m, being lower in the group of foreign women (15.5%) with an average height of 1.60 m.

As shown in Table 5, there is a correlation between the weight of the baby and the height of the mother, since the shorter the mother, the greater the probability of caesarean delivery, a fact that is true in the group of Spanish pregnant women.

In general, Spanish women had a mean gestation time of 40 ± 1.83 weeks, while the gestation time of foreign women was 39.72 ± 2.28 weeks. Moreover, Spanish women had more post-term pregnancies (>41 weeks gestation) than foreign women, while the latter had more preterm deliveries (<37 weeks gestation).

It is noteworthy that of the six women in the study who had preterm births, four corresponded to the group of foreign pregnant women. In addition, the data detailed in Table 4 reveal that foreign women had a higher number of instrumental or caesarean deliveries due to preterm pregnancies.

## 4. Discussion

This paper studies a cohort of Spanish and foreign pregnant women who followed their pregnancy at the Yepes Primary Care Centre, located in a rural area of the province of Toledo (Spain). The women followed the pregnancy protocol established by the obstetrics unit of the Castilla-La Mancha Health Service (SESCAM), on which the Yepes Health Centre depends.

The health professional’s supervision of the women during the three trimesters of gestation in relation to their state of health, analytical parameters and adherence to the 2000 kcal protocolised diet has resulted in the women achieving full-term deliveries and healthy newborns regardless of the culture or country of origin of the pregnant woman.

The gestational age of the sample in this study is in line with what the Spanish Society of Gynaecology and Obstetrics highlights as most likely to be associated with an adverse outcome during pregnancy [19,20,21,22].

As Illana et al. point out, among the reasons for delaying motherhood in our environment are job instability and job insecurity [23]. Not forgetting that the higher level of education attained by women in recent decades [24,25], together with the difficulty for young people to emancipate themselves from the parental home, are reasons for delaying motherhood [26,27,28,29]. This situation is also experienced by foreign women when they enter the labour market, a fact that even leads to the feminisation of certain jobs, a situation that is confirmed in the study, with an average gestational age of up to 36 years [24,25].

In relation to the level of purchasing power in this rural area, women carry out work activities with little training, as it is the partner who carries out their work outside the home [25,30]. While it is true that some research shows that foreign women carry out domestic tasks without outside help, this is associated with physical and psychological fatigue due to their long working hours [30,31,32]. In this sense, it can be affirmed that the social image of the Spanish pregnant woman in Yepes is that of a woman with secondary education, of medium socioeconomic level, economically independent and living with a partner. The social profile of the foreign pregnant woman in Yepes is that of a woman with secondary education, with a lower socio-economic level and economically dependent on her partner.

Several studies show that despite the HE received by the health professional, women with a low socioeconomic status may tend to consume a diet of lower nutritional quality [4,33,34,35]. These aspects are reflected in the type of delivery and weeks of gestation of the women in our study (38.55 weeks in the foreign group and 40.61 in the Spanish group), and even in the weight of the newborns born by caesarean section (foreigners 2911 g and Spaniards 3413 g).

It is interesting to analyse the variable maternal height as some studies consider this to be a very relevant aspect in the approach to childbirth [36,37], as pregnant women between 1.40 m and 1.56 m often tend to have cephalic-pelvic disproportion, give birth by caesarean section and even have low birth weight babies [36,37,38,39,40,41,42]. This fact could not be contrasted in our article as all the women in the sample were taller than 1.56 cm.

Since 1985, the WHO has been warning that the caesarean section rate worldwide is very high, and in this sense, the Global Maternal and Perinatal Health Survey and the Multi-country Maternal and Neonatal Health Survey insist on the need to reduce the global caesarean section rate [43,44,45]. In Spain, this rate has also increased [46,47]. Specifically, in 2018, in the reference hospital of the city of Toledo, caesarean sections accounted for 23.585% [48,49]. In our sample, 34.7% were caesarean deliveries, with the gestational age and weight of the newborn being of note.

The study warns that pregnant women tend to have an inadequate diet due to excessive consumption of convenience foods and pastries, with a low intake of dairy products, pulses, fruit, vegetables, meat and fish.

The literature shows that the gestational period in all women is a complex process that involves multiple changes in their lives, so it is very important that HE includes a healthy lifestyle [50,51,52,53] and diet [54,55,56,57,58,59]. The study warns that pregnant women tend to have an inadequate diet due to excessive consumption of convenience foods and pastries, with a low intake of dairy products, legumes, fruits, vegetables, meat and fish [54,55,56,57,58,59].

It is noted that the first trimester of pregnancy is a period of decreased appetite due to the discomfort experienced by some pregnant women in relation to hyperemesis gravidarum [60], a situation reflected in our study. In contrast, the perception of food in the second trimester is characterised by a pleasurable dimension, generally similar to that of the previous gestation [61,62,63]. The consumption of certain foods increased in both groups of pregnant women due to adequate HE such as eggs, even extrapolating this adequate intake to the different members of the family unit. However, consumption of fish and meat did not increase in the group of foreign women, possibly due to their economic dependence.

In both groups, consumption of convenience foods and cakes was very high, which may be related to cravings during the second trimester. In this regard, studies in pregnant women found that cravings, appetite and taste determine food choices, with pregnant women preferring diets [63,64,65].

Even so, certain studies emphasise that the risk of preterm birth is lower when the pregnant woman has a higher intake of vegetables, fruits, whole grains, nuts, legumes and seeds, with an exceptionally low intake of red meat, processed and fried foods [66,67,68,69].

Several studies indicate that, as a general rule, the dietary behaviour of foreigners in their home country was healthier than in the host country [70,71,72]. This may be due to changes in dietary behaviour over time, either because of cultural adaptation to the new country or because of the abuse of convenience foods, snacks and industrial pastries [73,74,75,76,77,78], a situation observed in Yepes’ foreign women.

Along the same lines, religion is a positive reinforcement for diet, as women strive to maintain their traditional customs and behaviours [79,80,81,82,83] and this situation was appreciated in our research, as Moroccan, Algerian, Tunisian and Syrian women, through their faith, achieved good results in their biochemical parameters.

### Strengths and Limitations

The results of the sample are limited to the rural environment of the municipality of Yepes, in the province of Toledo in Spain. Therefore, they cannot be generalised to all pregnant women living in Spain, whether native or foreign. The study is also limited by the presence of low-risk pregnancies.

Cultural and linguistic differences could represent a source of bias in the measures used, as the study only collected data from those women who speak Spanish, English and French, so the results cannot be generalised to women who do not speak these languages. It is possible that women with high social status did not participate in the study, as it is quite common for them to follow their pregnancies in private health care. Despite these potential biases, a diverse sample was recruited and maintained throughout the study, which is a strength of the study. Finally, this research was strengthened by prospective data collection, so that pregnancy outcomes were not biased.

## 5. Conclusions

The present study shows that HE has been fundamental for pregnant women in both groups to introduce healthy dietary modifications throughout the three trimesters of pregnancy, improving their health and favouring the correct development and growth of the new life. However, we believe that health promotion from the perspective of human development should take place prior to pregnancy, involving health services and the women themselves with the aim of improving the health conditions of pregnant women and their newborns. This would encourage, among other things, a healthier diet during pregnancy and the correct development of pregnancy and childbirth.

## Figures and Tables

**Table 1:** Socioeconomic characteristics of 194 women included in the study.

	Spanish	Foreign	*p*
*N*	121	73	
Socio-economic level according to average disposable income (*N*, %)			<0.001 ^2^
Low (<70%)	21 (17.4)	41 (56.2)	
Medium (70–125%)	58 (47.9)	23 (31.5)	
Medium-high (125–200)	26 (21.5)	6 (8.2)	
High (>200%)	16 (13.2)	3 (4.1)	
Economic dependence (*N*, %)			< 0.001 ^2^
Independent (woman with own salary)	66 (55.5)	18 (24.7)	
Dependent (woman without a salary)	53 (44.5)	55 (75.3)	
Education level (*N*, %)			0.0742 ^2^
Illiterate	0	3 (4.1)	
Primary School	32 (26.4)	21 (28.8)	
Secondary School	66 (54.5)	41 (56.2)	
Bachelor or higher	23 (19.0)	8 (11.0)	
Maternal age (years) mean ± SD	30.88 ± 5.88	30.47 ± 5.94	0.6401 ^1^
Live as a couple			0.7472 ^2^
Yes	114 (94.2)	70 (95.9)	
No	7 (5.8)	3 (4.1)	

Mean ± or frequency (%) values are shown. ^1^ *t*-Student test. ^2^ Chi-square test.

**Table 2:** Nutritional habits of 194 women included in the study.

Type of Aliments	Recommended Servings	Consume Recommended Amounts in T2 (*N*, %)		Consume Recommended Amounts in T3 (*N*, %)	
		Spanish	Foreign	Spanish	Foreign
Cereal	7	85 (70.8)	55 (75.3)	103 (85.12)	70 (95.89)
Pulse	7	63 (52)	58 (79.45)	79 (65.28)	67 (91.78)
Vegetables	4	77 (63.63)	47 (64.38)	116 (95.86)	71 (97.26)
Fruits	3	68 (56.2)	42 (57.53)	108 (89.25)	72 (98.63)
Dairy products	3	32 (26.44)	19 (26.02)	43 (35.53)	23 (31.5)
Meats	3	63 (52.06)	37 (50.68)	74 (61.15)	37 (50.68)
Fish	3	55 (45.45)	33 (45.2)	67 (55.37)	33 (45.2)
Eggs	2	21 (17.35)	15 (20.54)	33 (27.27)	30 (41)
Pre-cooked meals and pastries	1	90 (74.38)	53 (72.6)	53 (43.8)	37 (50.68)

**Table 3:** Biochemical parameters in pregnant women included in the study.

	Spanish	Foreign	*p*-Value ^1^
Total protein (mean ± SD) (g/dL)			
T1	7.00 ±0.37	7.25 ± 0.41	<0.001
T2	6.48 ± 0.35	6.64 ± 0.39	0.003
T3	6.23 ± 0.45	6.47 ± 0.37	<0.001
Albumin (mean ± SD) (g/dL)			
T1	4.43 ± 0.31	4.49 ± 0.28	0.175
T2	3.88 ± 0.32	3.94 ± 0.27	0.19
T3	3.69 ± 0.40	3.72 ± 0.22	0.5
Glucose (mean ± SD) (mg/dL)			
T1	81.49 ± 8.64	81.66 ± 8.58	0.894
T2	81.59 ± 9.86	81.66 ± 6.46	0.954
T3	75.31 ± 11.36	78.75 ± 10.86	0.042

Legends: T1: first trimester; T2: second trimester, and T3: third trimester of pregnancy. ^1^ *t*-Student test.

**Table 4:** Anthropometric characteristics and obstetric data of women included in the study.

	Spanish	Foreign	*p*-Value
Height (m)	1.61	1.62	0.2682 ^1^
Median [IR: Q3, Q1]	[1.58, 1.66]	[1.58, 1.68]	
Weight at T1 (Kg)	62	68	0.0262 ^1^
Median [IR: Q3, Q1]	[54.7, 71.75]	[60.0, 73.0]	
Weight at T3 (Kg)	71.5	73.5	0.1052 ^1^
Median [IR: Q3, Q1]	[63.00, 82.75]	[68.00, 72.25]	
BMI at T1 (Kg/m^2^)	23.99	25.05	0.0842 ^1^
Median [IR: Q3, Q1	[20.66, 27.17]	[22.24, 27.47]	
BMI at T3 (Kg/m^2^)	27.41	27.14	0.7412 ^1^
Median [IR: Q3, Q1]	[24.03, 30.71]	[25.00, 29.62]	
Weight gain T1-T3 (Kg)			0.1093 ^2^
(mean ± SD)	9.43 ± 4.20	8.42 ± 4.18	
Type of Birth (*N*, %)			0.5934 ^3^
Eutocic	85 (70.8)	55 (77.5)	
Instrumental	12 (10.0)	5 (7.0)	
Caesarean	23 (19.2)	11 (15.5)	
Newborn weight (Kg)	3.4	3.19	0.3612 ^1^
Median [IR: Q3, Q1]	[3.03, 3.61]	[3.00, 3.55]	

T1: first trimester of pregnancy, T3: third trimester of pregnancy. BMI: Body Mass Index; IR: Interquartile range. Mann–Whitney U, ^1^ Student’s *t*-test, ^2^ Chi-square, ^3^ test.

**Table 5:** Relationship between maternal height, birth weight and weeks of gestation.

Type of Delivery	Height of Mother (m)		Baby’s Weight (Kg)		Week of Gestation	
	Spanish	Foreign	Spanish	Foreign	Spanish	Foreign
Euthocic	1.625 ± 0.053	1.634 ± 0.071	3.309 ± 0.440	3.407 ± 0.477	39.92 ± 1.79	40.25 ± 0.93
Instrumental	1.628 ± 0.063	1.630 ± 0.071	3.196 ± 0.576	2.625 ± 0.991	40.17 ± 0.94	36.40 ± 4.93
Caesarean section	1.588 ± 0.051	1.600 ± 0.058	3.413 ± 0.465	2.911 ± 0.968	40.61 ± 1.23	38.55 ± 3.75

## Abbreviations

WHO	World Health Organization
HE	Health Education
SESCAM	Servicio de Salud de Castilla-La Mancha
